# Changes in patterns of retention in HIV care and antiretroviral treatment in Tanzania between 2008 and 2016: an analysis of routinely collected national programme data

**DOI:** 10.7189/jogh.09.010424

**Published:** 2019-06

**Authors:** Paul Mee, Brian Rice, Liis Lemsalu, James Hargreaves, Veryeh Sambu, Richelle Harklerode, Jim Todd, Geoffrey Somi

**Affiliations:** 1 The MeSH Consortium, London School of Hygiene and Tropical Medicine, London, UK; 2 Faculty of Epidemiology and Public Health, London School of Hygiene and Tropical Medicine, London, UK; 3 Faculty of Public Health and Policy, London School of Hygiene and Tropical Medicine, London, UK; 4 Institute of Family Medicine and Public Health, University of Tartu, Tartu, Estonia; 5 Department of Drug and Infectious Diseases Epidemiology, National Institute for Health Development, Tallinn, Estonia; 6 Strategic Information Unit, National AIDS Control Programme, Dodoma Tanzania; 7 University of California San Francisco, Global Health Sciences, San Francisco, California, USA

## Abstract

**Background:**

Tanzania is a high HIV burden country in Sub-Saharan Africa with 1.5 million people infected. Unless monitored and responded to, low levels of retention in care may lead to poor HIV associated clinical outcomes and an increased likelihood of onward viral transmission. Using routine data, we assessed changes in retention in care and on treatment for HIV over time in Tanzanian facilities, using the national care and treatment programme (CTC) database.

**Methods:**

Data were extracted from the CTC database and analysed using two approaches: a series of cross-sectional analyses for each calendar year between 2008 and 2016 to assess the changing characteristics of the population in care and on treatment, and, a longitudinal analysis using survival analysis methods for a series of cohorts representing i) all engaging in care and ii) all initiating treatment in each calendar year from 2008 to 2015. Multivariate analyses were carried out to explore the independent effect of calendar year when controlling for other factors.

**Results:**

The total number of individuals enrolled in care increased from 160 268 in 2008 to 548 296 in 2016. The percentage of the in-care population enrolled for more than 3 years increased from 9.9% in 2008 to 54.5% in 2016. The overall rates of retention in care were 80.9%, 57.3% and 45.4% at 12, 24 and 36 months respectively. The rates of retention on antiretroviral therapy (ART) ART at 12, 24 and 36 months after treatment-initiation were 83.9%, 64.0% and 53.5%. There were small but statistically significant differences in the retention rates between cohorts and evidence for a significant decrease in the rates of retention in the most recent years analysed.

**Conclusions:**

Data from Tanzania show that while the number of People Living with HIV (PLHIV) who were in care and monitored through the routine data system increased over time, the retention rates in care and treatment remained relatively stable. These rates were similar to other regional estimates. Systematic reviews of tracing studies indicate that mortality among those lost to follow up has decreased over time, partly underpinned by an increase in the numbers transferring between clinics. True retention rates may therefore be higher than we report here, and this underpins the need for data systems that can track patients between clinics.

The first cases of HIV in Tanzania were identified in 1983 and by 1986 new cases were reported in all regions of the country [1]. By 1998 there were estimated to be 1.4 million people living with HIV (PLHIV) in Tanzania, increasing to 1.5 million by 2017 [2]. In 2004, a national HIV care and treatment programme (CTC) was established to provide HIV services to PLHIV. The CTC programme monitors the provision of, and adherence to, antiretroviral therapy (ART). High levels of adherence have been shown to improve health outcomes, and high coverage in the surrounding community reduces the risk of onward transmission [3].

In order for the population level roll-out of ART to achieve its full potential; the goal of treatment programmes should be that each HIV positive individual knows their status, enters care, initiates ART when eligible and is subsequently retained in care and remains adherent to treatment. Treatment targets such as the UNAIDS 90:90:90 seek to establish benchmarks based on these targets against which the performance of national HIV treatment programmes may be assessed [4,5]. In 2012 in Tanzania, it was estimated that 51% of PLHIV knew their HIV sero-status and 31% received ART [2,6]. A study of 55 000 adults enrolling in HIV care in Tanzania between 2005 and 2011 showed that among persons assessed for ART eligibility, 53% were eligible for treatment, of these 70% initiated treatment and of those starting ART 75% were retained on ART 12 months after commencing treatment. [7]. The definition of eligibility was based on the national guidelines at the time which largely reflected the most recent WHO recommendations. In 2013, Tanzania adopted the 2012 World Health Organization (WHO) recommendation that PLHIV with a CD4 count less than 350 cells/ml be initiated on ART [1]. In 2017, Tanzania adopted the updated WHO guidance that ART should be initiated in all HIV positive individuals regardless of CD4 count [8]. The routine use of viral load monitoring was introduced in the Tanzanian national HIV programme in 2016.

Three systematic reviews of studies of sub-Saharan African HIV treatment cohorts indicate that there is substantial attrition of patients at each step along the HIV treatment cascade [9-11]. Of those initiated on ART in Tanzania from 2003- 2007, 58% were retained after 36 months. An increased hazard for attrition was observed in the first 6 months after starting ART and between years 3 and 4 while on treatment [12].

In this study we assess whether levels of retention in care and on treatment have changed over time as the coverage of the Tanzanian national care and treatment programme has expanded.

## METHODS

### Data management

Every health facility in Tanzania, that has an HIV care and treatment clinic (CTC), uses the standard, paper-based, patient management forms provided by the National AIDS Care Programme (NACP). These include information on symptoms, test results (CD4 count), signs (WHO clinical stage), ART regimens prescribed with the initiation and switching dates, and mortality-related data. At enrolment into CTC, all patients are assigned a unique CTC identification number. Patients re-engaging with care after an interruption of treatment or transferring between health facilities are often assigned a new unique CTC identification number. At clinics with access to computers, all historic and current data are captured in a facility-level database standardized by NACP. Regular quality control checks are carried out to verify the accuracy of the data capture process. Periodically, data without patient names, are extracted from the local databases into the national CTC-3 database which contains records for approximately 67% of all patients enrolled in HIV care in the country [13].

This analysis used extracted data from patients, aged over 15 years, collected since 2008 when the ART program was expanded to cover the entire country. Data cleaning was carried out to ensure data fell within reasonable ranges and was internally consistent. The date of patient enrolment was defined as the earliest recorded visit to CTC, and the ART initiation date was the first visit where ART was prescribed. Date of birth was defined based on a self-report by the patients and confirmed at subsequent clinic visits. According to NACP programme guidelines a patient is defined as lost to follow-up if they fail to attend for a follow-up within 90 days of their next scheduled appointment. Where appointment dates were not recorded the dates were imputed based on the programme’s standard visit frequency schedules. The CTC-3 database was estimated in March 2015 to have captured 67% (464 813 out of a best estimate of 690 944) of those persons in receipt of treatment nationally [13].

### Cross-sectional data analysis

The study population for the cross-sectional analysis comprised all individuals with a visit between January 1^st^ 2008 and December 31^st^ 2016. For each calendar year, data for all patients presenting to the CTC, were aggregated. For each individual, the time in care was calculated as the elapsed time from enrolment to the last care visit in a particular calendar year. Length of time on ART was defined, for those who commenced treatment, as the time from ART initiation to the last visit at which ART was prescribed in that year. The percentages of the total population in care and total population on ART for <1 year, 1 to 2 years, 2 to 3 years and greater than 3 years were calculated for each calendar year. Visits in the first 90 days after enrolment were excluded.

### Longitudinal analyses

In the longitudinal analysis we included all patients enrolled from 1^st^ January 2008, and data on all their visits up to 31^st^ December 2016. Clinics were only included if visits were recorded in each year subsequent to the year they first submitted data, this prevented patients being misclassified as lost to follow-up due to a clinic failing to submit data in a particular year. Due to changes in administrative boundaries in Tanzania a sub-set of clinics were assigned new identifier codes in 2011. As a result, in this study, data from these clinics submitted before the code re-assignment were excluded from the analysis. To assess any resultant bias, the data was stratified by year of first data submission and the rates of loss to follow-up from care before and after 2011 compared.

Following this, the overall population was divided into those excluded from and included in the final study population. Each group was stratified by gender, age, year of enrolment and type of facility at which enrolment occurred. Comparisons were made to see if the two groups differed systematically.

Due to the incompleteness of data on mortality whilst in care we combined all reasons for disengagement as a single endpoint which occurred at 90 days after the last scheduled visit, as defined in the NACP guidelines. Cohorts were created comprising all those engaging in care or initiating treatment in each calendar year from 2008 to 2015. As there is a time lag between enrolment and treatment initiation it is possible for the numbers starting on treatment in a particular year to be greater than the number enrolling in care in the same year. Separate analyses were carried out for retention in care, commencing at the date of engagement in care and retention on treatment, commencing on the date ART was first prescribed. In each case, sub-analyses stratified by gender and age in four groups (15 to 24, 25 to 34, 35 to 49, and 50 and over) were subsequently carried out. Bivariate and multivariate Cox regression analyses were carried out to assess whether key variables (calendar year, gender, age and the type of health facility) were independently and jointly associated with the risk of attrition from care and treatment.

### Ethics

The study and analyses of the CTC data are covered by ethical approval from National Institute for Medical Research NIMR/HQ/R.8a/Vol. IX/2097.

## RESULTS

**Figure 1:**
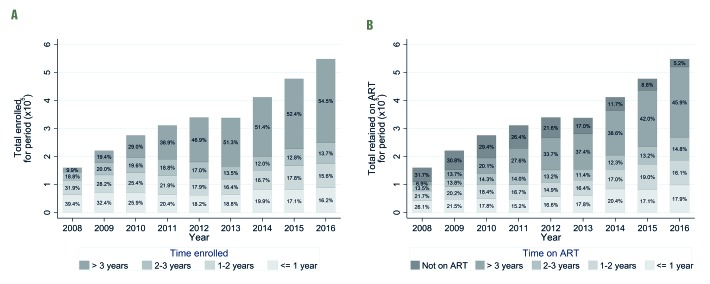
**Panel A.** Cross-sectional analysis of length of time enrolled in care (percentage and absolute number of patients) by year. **Panel B.** Cross-sectional analysis of the length of time on antiretroviral therapy (ART) (percentage and absolute number of patients) by year.

The total number of individuals with a CTC visit in a particular year increased from 160 268 in 2008 to 548 296 in 2016. In 2008, 39.4% of those in care had been enrolled for less than 1 year, this had decreased to 16.2% by 2016, whilst the percentage enrolled for more than 3 years increased from 9.9% to 54.5% over the same period (**Figure 1**, panel A).

The percentage of those in care who had been on antiretroviral treatment for less than 1 year decreased from 26.1% in 2008 to 17.9% in 2016, whilst the percentage who had been on treatment for more than 3 years increased from 6.9% to 45.9% over the same period. The percentage of those in care not yet on treatment decreased from 31.7% to 5.2% between 2008 and 2016 (**Figure 1**, panel B).

The total study population, based on the first care visit, consisted of 743 801 individuals of which 90 596 were excluded as the clinic at which they were first enrolled failed to provide data in at least one year after the first year it submitted data to the CTC-3 database (**Table1**). The comparison between those included in and excluded from the longitudinal cohort showed that those excluded were more likely to have been enrolled in the earlier years of follow-up, 10.4% of those included were enrolled in 2008 compared to 28.5% of those excluded and 15.0% of those included were enrolled in 2015 compared to 4.0% of those excluded (**Table 1**), there was strong evidence (*P* < 0.001) that this difference was statistically significant.

**Table 1:** Characteristics of persons included in and excluded from annual longitudinal cohorts 2008 to 2015

	Included in the longitudinal cohort N (%)*	Excluded from the longitudinal cohort N (%)*	*P*-value†
**Gender:**			
Male	208 004 (31.8%)	29 137 (32.2%)	0.157
Female	445 194 (68.2%)	61 458 (67.8%)	
Missing	7 (0.0%)	1 (0.0%)	
**Age at enrolment (years):**			
15-24	75 596 (11.6%)	9276 (10.2%)	<0.001
25-34	232 794 (35.6%)	33 854 (37.4%)	
35–49	263 615 (40.4%)	37 163 (41.0%)	
>49	81 200 (12.4%)	10 303 (11.4%)	
**Year of enrolment:**			
2008	67 840 (10.4%)	25 809 (28.5%)	<0.001
2009	78 805 (12.1%)	22 476 (24.8%)	
2010	78 395 (12.0%)	18 878 (20.8%)	
2011	69 337 (10.6%)	13 572 (15.0%)	
2012	70 336 (10.8%)	1522 (1.7%)	
2013	87 761 (13.4%)	1624 (1.8%)	
2014	102 611 (15.7%)	3076 (3.4%)	
2015	98 120 (15.0%)	3639 (4.0%)	
Type of health care facility			
Dispensary	136 528 (20.9%)	11 954 (13.2%)	<0.001
Health Centre	224 325 (34.3%)	16 522 (18.2%)	
Hospital	284 430 (43.5%)	60 580 (66.9%)	
Missing	7922 (1.2%)	1540 (1.7%)	
**Total**	**653** **205**	**90** **596**	

*The longitudinal cohort was limited to those enrolled in 2008 to 2015. Where a clinic failed to submit data in at least one year between the first year of data submission and 2015, data from that clinic was excluded from the analysis cohort.

†The χ2 tests the probability that the difference in proportions between those excluded and included in the cohort might have occurred by chance.

Those included in the longitudinal cohort were more likely to have been initially enrolled at a dispensary (20.9% of those included compared to 13.2% of those excluded), or health centre (34.3% of those included compared to 18.2% of those excluded). Those excluded from the cohort were more likely to have been enrolled in a hospital (66.9% compared to 43.5% of those included), again there was strong evidence (*P* < 0.001) for the statistical significance of these differences. (**Table 1**).

### Retention in care

The overall percentages retained in care after enrolment were 80.9% (95% confidence interval (CI) = 80.6-81.1), 57.3% (95% CI = 56.9-57.6) and 45.4% (95% CI = 45.1-45.8) at 12, 24 and 36 months respectively (**Table2**).

**Table 2:** Comparison of total enrolled in care in each annual cohort and number retained at 12, 24 and 36 months*

		Percentage retained in care at time (95% confidence intervals)		
**Year of enrolment in care**	**Total enrolled in care**	**12 months**	**24 months**	**36 months**
**2008**	67 840	82.8 (82.5-83.0)	59.2 (58.8-59.5)	46.7 (46.3-47.1)
**2009**	78 805	81.5 (81.2-81.8)	56.9 (56.5-57.2)	44.5 (44.2-44.9)
**2010**	78 395	79.8 (79.5-80.0)	55.4 (55.1-55.8)	44.2 (43.9-44.6)
**2011**	69 337	80.2 (79.9-80.5)	57.2 (56.8-57.5)	45.6 (45.2-45.9)
**2012**	70 336	80.6 (80.3-80.9)	57.2 (56.9-57.6)	45.1 (44.8-45.5)
**2013**	87 761	80.5 (80.3-80.8)	57.5 (57.2-57.8)	46.3 (46.0-46.7)
**2014**	102 611	80.4 (80.1-80.6)	57.7 (57.4-58.0)	–†
**2015**	98 120	81.5 (81.2-81.7)	–†	–†
**Overall**	**653** **205**	**80.9 (80.6-81.1)**	**57.3 (56.9-57.6)**	**45.4 (45.1-45.8)**

*An expanded version of this table in which the study population is stratified by age and sex is included in Table S1 in **Online Supplementary Document**.

†These percentages were not included in the table as some of the cohort have not had the opportunity to accrue sufficient follow-up time prior to the censoring date of 31 September 2017.

**Figure 2:**
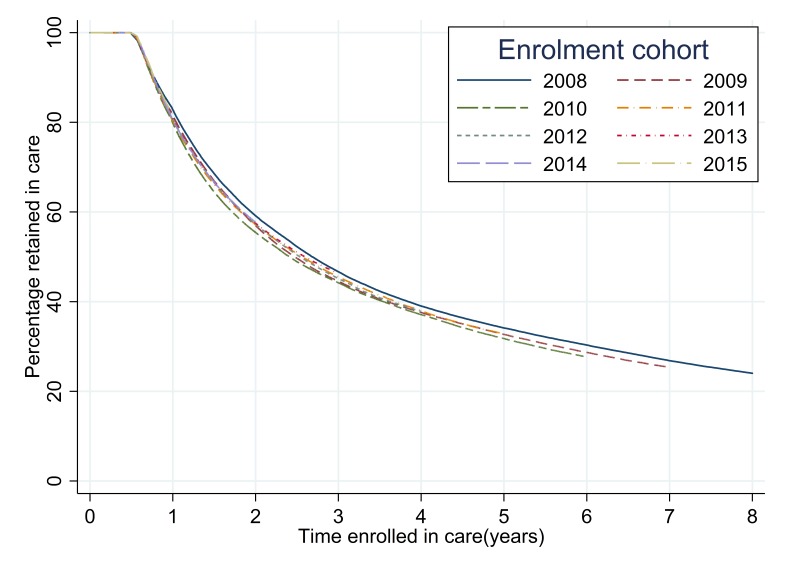
Time to event analysis of retention in care following enrolment for each successive annual enrolment cohort.

The analysis of retention in care for each successive annual cohort (**Table 2****,** **Figure 2**) showed that there were only small changes between cohorts in the percentage retained for each time period up to 36 months. The highest level of retention in care was seen for those enrolled in 2008, where 46.7% (95% CI = 46.3-47.1) were retained in care at 36 months after initial enrolment, the lowest levels of retention in care at 36 months were seen for the 2010 enrolment cohort, of whom 44.2% (95% CI = 43.9-44.6) were retained. The levels of retention at 36 months subsequently increased to 46.3% (95% CI = 46.0-46.7) for the 2013 enrolment cohort. Retention at 24 months ranged from 59.2% (95% CI = 58.8-59.5) in 2008 to 55.4% (95%CI = 55.1-55.8) in 2010. At 12 months after enrolment, retention ranged from 82.8% (95%CI = 82.5-83.0) in 2008 to 79.8% (95% CI = 79.5-80.0) in 2010. The log-rank test for the equality of survivor functions for each enrolment cohort had a *P*-value of <0.0001 indicating that there were significant differences between the retention curves for each successive cohort.

Retention in care was consistently higher for females than males, this was seen for all enrolment cohorts at 12, 24 and 36 months of follow-up (Table S1a in **Online Supplementary Document**). As an example, for the 2013 cohort the percentages of females retained at 12, 24 and 36 months were 81.3% (95% CI = 81.0-81.6), 58.1% (95% CI = 57.7 -58.5) and 46.6% (95% CI = 46.2-47.0) respectively. For males the comparative figures were 78.9% (95% CI = 78.5-79.4), 56.2% (95% CI = 55.6-56.8) and 45.7% (95% CI = 45.1-46.2). The difference in retention in care between males and females decreased over time. In 2008, 84.1% (95% CI = 83.8–84.4) of females were retained in care at 12 months compared to 79.9% (95% CI = 79.3-80.4) of males. By 2015, the corresponding figures were 81.8% (95% CI = 81.5-82.1) for females and 80.9% (95% CI = 80.4-81.3) for males. (Table S1a in **Online Supplementary Document**).

The highest levels of retention in care were seen for those aged between 35 and 49. Levels of retention decreased for those in successively lower and higher age groups. This pattern was seen consistently for each enrolment cohort and at each retention milestone (Table S1b in **Online Supplementary Document**). There was also evidence for an increased difference over time in the levels of retention in care for those aged 15-24 compared to those in older age groups. The difference was principally due to decreases in the percentage retained in the youngest age group. In 2008 81.2% (95% CI = 80.3-82.1) of those aged 15-24 were retained in care at 12 months compared to 82.3% (95% CI = 81.4-83.2) of those aged greater than 49. By 2015 the percentages retained were 76.5% (95% CI = 75.8-77.2) for those aged 15-24 and 83.3% (95% CI = 82.7-83.9) for those aged greater than 49 (Table S1b in **Online Supplementary Document**).

The bivariate Cox regression analysis (**Table 3**) indicated that the hazard rates for loss to follow-up from care were greater for all cohorts after that enrolled in 2008, however there was no evidence for a sustained increase or decrease in the rate over time. These associations were maintained with only small numerical differences in the adjusted hazard rate ratios and confidence intervals in the multivariate analysis. There was also evidence from the multivariate analysis that retention in care was greater for those enrolled in dispensaries than those enrolled in health centres and hospitals (**Table 3**).

**Table 3:** Bivariate and multivariate analysis using Cox regression comparing rate of loss to follow-up from enrolment in care

Category	Bivariate analysis		Multivariate analysis	
Rate ratio*	**95% CI**	Rate ratio*	**95% CI**
**Year of enrolment:**				
2008	1	–	1	–
2009	1.05	1.04-1.07	1.07	1.06-1.09
2010	1.09	1.08-1.10	1.13	1.12-1.15
2011	1.06	1.05-1.07	1.10	1.09-1.12
2012	1.06	1.04-1.07	1.11	1.10-1.13
2013	1.04	1.02-1.05	1.10	1.08-1.11
2014	1.05	1.04-1.07	1.13	1.11-1.14
2015	1.03	1.01-1.04	1.10	1.09-1.12
**Sex:**				
Male	1	–	1	–
Female	0.94	0.93-0.94	0.92	0.91-0.93
**Age at enrolment (years):**				
15-24	1	–	1	–
25-34	0.79	0.78-0.80	0.76	0.75-0.77
35-49	0.66	0.65-0.66	0.59	0.59-0.60
>49	0.67	0.66-0.68	0.57	0.56-0.57
**Type of health care facility:**				
Dispensary	1	–	1	–
Health centre	1.03	1.02-1.04	1.04	1.03-1.05
Hospital	1.05	1.04-1.05	1.07	1.06-1.08

CI – confidence interval

*Compares the rates loss to follow-up after enrolment in care between the baseline category and each subsequent stratum.

### Retention on antiretroviral treatment

The overall percentages of the study population retained on ART at 12, 24 and 36 months after treatment-initiation were 83.9% (95% CI = 83.6-84.2), 64.0% (95% CI = 63.7-64.4) and 53.5% (95% CI = 53.0-53.9) (**Table 4**).

**Table 4:** Comparison of total initiated on antiretroviral therapy (ART) in each annual cohort and number retained at 12, 24 and 36 months

		Percentage retained on ART at time (95% confidence intervals)		
**Year of ART initiation**	**Total initiated on ART**	**12 months**	**24 months**	**36 months**
**2008**	34 584	84.6 (84.3-85.0)	65.5 (65.1-66.0)	53.5 (53.0-54.0)
**2009**	47 869	84.4 (84.1-84.8)	64.3 (63.9-64.8)	52.3 (51.8-52.7)
**2010**	52 041	83.0 (82.7-83.3)	63.0 (62.6-63.4)	52.3 (51.8-52.7)
**2011**	51 780	84.4 (84.1-84.8)	65.5 (65.1-65.9)	54.4 (54.0-54.9)
**2012**	66 432	85.1 (84.9-85.4)	66.0 (65.7-66.4)	54.4 (54.0-54.7)
**2013**	83 723	84.4 (84.2-84.7)	64.5 (64.1-64.8)	53.6 (53.2-53.9)
**2014**	107 030	82.7 (82.5-83.0)	61.7 (61.4-62.0)	–*
**2015**	100 616	83.6 (83.4-83.9)	–*	–*
**Overall**	**544** **075**	**83.9 (83.6-84.2)**	**64.0 (63.7-64.4)**	**53.5 (53.0-53.9)**

ART – antiretroviral therapy

*Insufficient follow-up time for 24/36 month retention to be calculated

**Figure 3:**
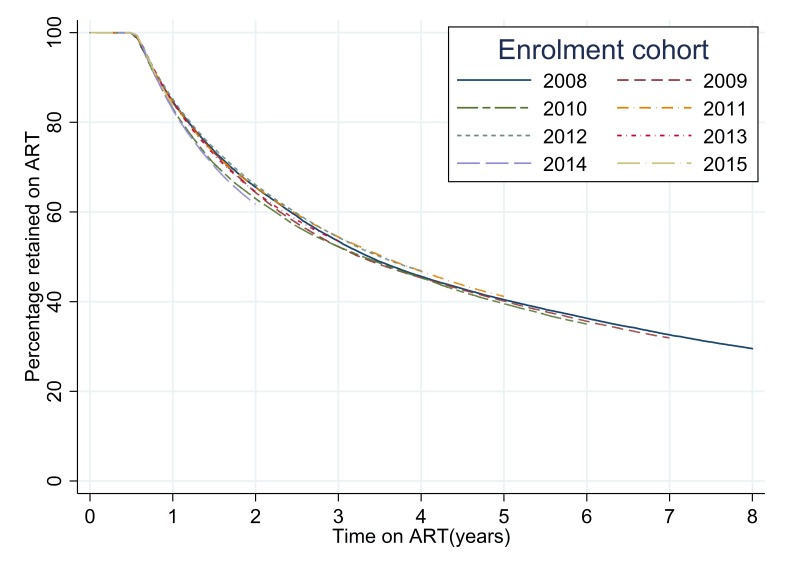
Time to event analysis of retention on antiretroviral therapy (ART) for each successive recruitment cohort.

The analysis of retention on ART for each successive cohort revealed patterns that were similar to those seen from the analysis of patterns of retention in care after enrolment (**Table 4**, **Figure 3**). The highest overall levels of retention were seen for the 2012 ART initiation cohort with the percentages retained on treatment at 12, 24 and 36 months after initiation of 85.1% (95% CI = 84.9-85.4), 66.0% (95% CI = 65.7-66.4) and 54.4% (95% CI = 54.0-54.7). The lowest levels of retention on ART were seen for the 2014 cohort with retention at 12 and 24 months of 82.7% (95% CI = 82.5-83.0) and 61.7% (95% CI = 61.4-62.0). The log-rank test for the equality of survivor functions for each ART retention cohort had a *P*-value of <0.0001 indicating that there were significant differences between the retention patterns for each successive cohort (Table S2a in **Online Supplementary Document**).

The percentages of males and females retained on ART converged over time; in 2008 85.8% (95% CI = 85.4-86.2) of females were still on treatment at 12 months compared to 82.3% (95% CI = 81.7-83.0) of males, whereas by 2015 the corresponding percentages were 83.7% (95% CI = 83.5-84.0) for females and 83.3% (95% CI = 82.9-83.8) for males. Please see Table S2a in **Online Supplementary Document**).

Retention improved with increasing age strata up to those aged 35–49 years and then decreased for those aged 50 years and older at enrolment (Table S2b in **Online Supplementary Document**). There was also evidence for a significant decrease in the percentages retained on ART for those aged 15-24 between 2008 and 2015 which was not seen for those in older age groups. For the 2008 cohort 82.9% (95% CI = 81.4-84.2) of those aged 15-24 were retained on ART at 12 months compared to 85.3% (95% CI = 84.8-85.8) of those aged 35-49. By comparison for the 2015 cohort 77.5% (95% CI = 76.8-78.3) of those aged 15-24 were retained in care at 12 months compared to 85.8% (95% CI = 85.5-86.2) of those aged 35-49 (Table S2b in **Online Supplementary Document**).

The bivariate Cox regression analysis comparing the rates of default from ART treatment (**Table 5**) showed that for all years other than 2011 and 2012 the hazard rate for default from treatment was higher than that for the 2008 enrolment cohort. The effect was particularly marked for the 2014 and 2015 cohorts, for example for those starting treatment in 2014, the hazard rate ratio compared to 2008 was 1.15 (95% CI = 1.13-1.17). In the multivariate analysis controlling for the effect of sex, age and type of health care facility the adjusted hazard rate ratios for default from ART treatment were greater than 1 in all years compared to 2008 and a larger increase in 2014 and 2015 was seen. There was also some evidence from the multivariate analysis that retention on ART was higher for those enrolled at dispensaries compared to those enrolled at health centres and hospitals (**Table 5**).

**Table 5:** Bivariate and multivariate analysis using Cox regression comparing rate of loss to follow-up after antiretroviral therapy (ART) initiation

Variable and category	Bivariate analysis		Multivariate analysis	
Rate ratio*	**95% CI**	Rate ratio*	**95% CI**
**Year of enrolment:**				
2008	1	–	1	–
2009	1.02	1.01-1.04	1.04	1.02-1.06
2010	1.05	1.03-1.06	1.08	1.06-1.10
2011	0.99	0.98-1.01	1.03	1.01-1.05
2012	0.98	0.97-1.00	1.03	1.01-1.05
2013	1.02	1.00-1.03	1.07	1.05-1.09
2014	1.15	1.13-1.17	1.21	1.19-1.23
2015	1.13	1.11-1.15	1.19	1.17-1.21
**Sex:**				
Male	1	–	1	–
Female	0.93	0.92-0.93	0.90	0.89-0.91
**Age at enrolment (years):**				
15-24	1	–	1	–
25-34	0.74	0.73-0.75	0.72	0.71-0.73
35-49	0.59	0.59-0.60	0.55	0.54-0.55
>49	0.61	0.60-0.62	0.52	0.51-0.53
**Type of health care facility:**				
Dispensary	1	–	1	–
Health centre	0.99	0.98-1.00	1.01	1.00-1.03
Hospital	0.98	0.97-0.99	1.03	1.02-1.04

CI – confidence interval

*Compares the rates of default from treatment between the baseline category and each subsequent stratum.

## DISCUSSION

In this study we assessed changes over time in the recruitment and retention of individuals in the Tanzanian national HIV care and ART programme. We compared the results from cross-sectional analyses with those from longitudinal analyses, which allowed the rates of transition between enrolment in care or initiation on ART and loss to follow-up to be analysed. The population in care and on treatment at clinics using the routine data system increased rapidly. Between 44% and 47% of individuals were retained in care 3 years after enrolment and between 52% and 54% of those starting treatment were retained on ART 3 years after treatment initiation.

There was a small but statistically significant decrease in the percentages of individuals retained in care between the 2008 and 2015. This decrease occurred as the total numbers engaging in care and initiating on treatment increased. As would be expected, attrition from care and treatment continued beyond the end of year 3. However, the rate of attrition was lower after this time period. This is to be expected as those retained beyond 3 years would represent a subset of the in-care population who have become skilled at managing their own engagement with care. There was evidence of a significant increase in the rates of default both from engagement with care and from treatment for those starting treatment in 2014 and 2015. This will need to be closely monitored in future years.

Clinics which submitted first submitted data in the early years of follow-up were more likely to have their data excluded from the longitudinal cohort as the likelihood of them not submitting data in any one particular year was greater than that for those clinics first submitting data later. This would be the most likely explanation for the finding that individuals enrolling earlier in the study were more likely to be excluded than those enrolling later.

The overall retention rates we report are somewhat lower than shown by a meta-analysis assessing levels of retention for patients on ART programmes in low and middle-income countries between 2008 and 2013 [14] which found that in Africa 65% of those initiating treatment were retained at 36 months with the highest loss to follow up in the first 6 months after treatment initiation.

Aggregate summaries presenting characteristics of the current in-care population such as those presented in the cross-sectional analyses in this paper are typical of the data seen by HIV programme managers. Our results showed that with each successive year the percentage of the overall study population who had been in care for greater than 3 years increased from close to 10% in 2008 to over 50% in 2016. Similarly, the percentage who had been on ART for greater than 3 years increased in each successive year from around 9% in 2008 to over 45% in 2016. Such changes might be interpreted as indicating that levels of long-term retention in care and on treatment were improving over time. However, the longitudinal cohort analyses present a somewhat different picture with statistically significant but small and variable changes in the rates of loss to follow-up and on treatment over time. Stratified analyses revealed that retention in care and on treatment was consistently worse for males enrolled in the programme than females. The differences in retention between the genders did however decrease over time, this is likely to be due to the fact that females had earlier access than males to effective HIV prevention and treatment information through their engagement with Antenatal care programmes [15]. This contrasts with a review of retention in care in CDC funded programmes in six countries in Eastern and Southern Africa between 2004 and 2012 that found no difference in the level of retention between men and women [16]. An analysis of data from 15 ART treatment programmes in lower income settings in Africa, Asia and South America similarly found no evidence of differential loss to follow-up by sex [17].

The patterns of change with age are interesting, levels of retention in care and on treatment improved with increasing age up to the age of 50 and then showed evidence for poorer retention for those who initiate care aged over 50. This is likely to be due in part to the fact that our endpoint was a combined measure of death and loss to follow-up and the rates of mortality would be expected to be higher in the oldest age groups. The decrease in levels of retention in care and on treatment over time among those aged less than 25 is a cause for concern which requires more in-depth investigation.

The analysis showed evidence for higher levels of retention in care and on treatment for those enrolled at dispensaries compared to those enrolled at hospitals and health centres. This may be due to the patients at the higher level facilities having more complex medical needs however it may also provide further evidence for the advantages associated with the ‘down-referral’ of patients to health facilities which are closer to where they live that have been previously identified [18].

This analysis is subject to several limitations. Data was only available from health facilities with the infrastructure to collect patient-level electronic data. An estimated 67% of those enrolled into HIV care nationally were enrolled at such facilities. A consequence of this was that individuals attending hospitals were more likely to be included in the CTC-3 database than individuals attending health centres and dispensaries. Although a previous analysis found there were no significant differences in patient outcomes when the data was stratified by type of facility [12], there remains the possibility that in this updated data set there are differences in the clinical characteristics between those included and excluded. It is not possible to conclude whether this would lead to an under or over-estimation of the true rate of loss to follow-up compared to all individuals in HIV care. It may be that the larger better equipped health facilities tend to be located further from an individual’s home leading to a greater level of loss to follow-up due to travel distance presenting a greater barrier to accessing health care [17,19,20]. Alternatively, the increased quality of care offered at these facilities may provide sufficient incentive to overcome such barriers [21].

The longitudinal analysis only included clinics contributing data in each year of follow-up after their first submission to CTC-3. This led to those enrolled in the earlier calendar years and those enrolled in smaller medical facilities having a greater likelihood of being excluded. Patients re-engaging with care after a treatment interruption or transfer between health facilities were usually assigned a new CTC identification number, this is likely to lead to an overestimation of the percentages either lost to follow-up or defaulting from treatment. Additionally, an administrative change in 2011 led to some clinics being assigned new codes in that year. When a comparison was made between clinics first submitting data in 2010 and 2012, the hazard rate ratios for loss to follow-up from care compared to 2005 were identical (hazard rate ratio = 0.99, 95% CI = 0.97-1.01) suggesting that this change had not introduced a significant bias in our analysis. (Table S3 in **Online Supplementary Document**).

Due to the difficulty in tracing treatment outcomes for those who were lost to follow-up, the mortality rate for patients in care in the CTC database was likely to be an underestimate of the true figure. For this reason, we were not able to distinguish those who had defaulted from treatment from those who had died whilst engaged in HIV care. The lack of linked data on HIV testing prevented us estimating the first 90 in the UNAIDS 90:90:90 indicators [4] (percentage of those infected who were diagnosed and know their HIV status), or the second 90 (percentage of those who know their HIV status who are on treatment). Additionally, we were not able to make a direct estimate of the third ‘90’ (percentage on treatment with suppressed viral loads) due to a lack of either viral load or CD4 count data from the follow-up period. Data on the individual’s WHO clinical stage [22] was available at the majority of visits, however an analysis of this showed that there was a lack of evidence of a transition between stages after ART initiation, limiting its usefulness as a proxy indicator for changes in viral load. The lack of CD4 data at treatment initiation, limited the ability to assess the eligibility criteria for ART.

Within the CTC data it was not possible to link separate episodes of engagement in care for the same individual due a lack of unique personal identifiers, thus in this analysis we were unable to explore the patterns of engagement and disengagement with care. For the same reason we were unable to explore the extent to which patients may enter the cascade at intermediate stages rather than by progressing from pre-ART care to ART initiation, the so-called ‘side-door’ into the HIV care cascade [23].

A systematic review of data from African HIV treatment programmes found that mortality among patients lost to follow-up who could be traced decreased over time, dropping from around 56% in 2003 to 24% in 2011 [24]. This is likely to be related at least in part to increases in the CD4 count threshold for treatment initiation. The review also found that over the same period, the proportion of patients lost to follow-up who were found to have transferred to another facility, so called silent transfers, had increased albeit without crossing the threshold for statistical significance [24]. If these trends were a reflection of the underlying patterns of engagement with care in Tanzania, the apparently small changes in loss to follow-up in our study may be indicative of an overall improvement in retention in the national programme. This highlights the need for the CTC programme to develop a system of unique personal identifiers which can be carried with an individual when they transfer between clinics enabling the silent transfers to be properly accounted for and de-duplicated in the national CTC-3 database.

The WHO “Test and Treat” strategy, in which all HIV positive patients are initiated on ART regardless of CD4 count was adopted in Tanzania in 2017 [8]. Ongoing monitoring will be needed in order to assess the impact of this new policy on patterns of retention in care and on treatment.

Both cross-sectional analyses and longitudinal analyses are important to health policy makers, and analyses similar to those reported in this paper have been undertaken by NACP personnel in the past. Repeated, consistent analyses will not only assist policy makers with relevant results, but also encourage better data collection and reporting from the health facilities.

## CONCLUSIONS

This study provides important insights into programme performance and the differences in engagement with care and subsequent retention in care and on treatment between genders and by age. An important finding is that despite large expansions in the HIV care programme and the implementation of earlier treatment initiation, there has not been a large decrease in the percentage of those enrolled who are retained in care.

Previous studies have identified the advantages of developing longitudinal care cascade models that enable a fine-grained insight to be gained into relative retention for different population sub-groups. The alternative cross-sectional approach can lead to substantial biases in the estimation of losses along the cascade [25,26]. The longitudinal analyses additionally allow an exploration of the transitions between stages in the care cascade, both in terms of the percentages of the original care cohort progressing and importantly the times for transition and the losses from the cascade that occur during the transition. Inferences about transition probabilities can be drawn from cross-sectional cascades but these rely on the assumption that current transition probabilities are the same as those at a previous point when the overall composition of the population in care was different [26]. As increasing numbers of individuals need to be maintained on treatment in resource-limited countries, it is essential that better use is made of routinely collected clinical data in order to enable programme managers to evaluate and improve key metrics of programme performance in a timely manner.

## Additional Material

Online Supplementary Document

## References

[1] National AIDS Control Programme The United Republic of Tanzania Ministry of Health and Social Welfare - Implementation of HIV/AIDS Care and Treatment Services in Tanzania - Report Number 3 http://www.nacp.go.tz/site/download/bookreport3.pdf Accessed 20-Jul-20182013.

[2] UNAIDS AIDS Info. 2016. http://aidsinfo.unaids.org/ Accessed 14-Sept-2018.

[3] Tanser F, Bärnighausen T, Grapsa E, Zaidi J, Newell M-L (2013). High coverage of ART associated with decline in risk of HIV acquisition in rural KwaZulu-Natal, South Africa.. Science.

[4] UNAIDS. 90-90-90: an ambitious treatment target to help end the AIDS epidemic. Report. Geneva: UNAIDS; 2014.

[5] UNAIDS. Fast-track: ending the AIDS epidemic by 2030. Report. Geneva: UNAIDS; 2014.

[6] Staveteig S, Croft TN, Kampa KT, Head SK (2017). Reaching the ‘first 90’: Gaps in coverage of HIV testing among people living with HIV in 16 African countries.. PLoS One.

[7] GuidelinesMcNairy ML Lamb MR Abrams EJ Elul B Sahabo R Hawken MP Use of a comprehensive HIV care cascade for evaluating HIV program performance: findings from 4 sub-Saharan African countries. J Acquir Immune Defic Syndr 2015 70 e44 51 10.1097/QAI.0000000000000745 26375466 PMC5094356

[8] Tanzania Ministry of Health, Community Development, Gender, Elderly and Children. National AIDS Control Programme. National Guidelines for the Management of HIV and AIDS - Sixth Edition - October 2017. Available: http://nacp.go.tz/site/download/NATIONAL_DECEMBER_2017.pdf Accessed:14-Sept-2018.

[9] Rosen S, Fox MP (2011). Retention in HIV care between testing and treatment in sub-Saharan Africa: a systematic review.. PLoS Med.

[10] Kranzer K, Govindasamy D, Ford N, Johnston V, Lawn SD (2012). Quantifying and addressing losses along the continuum of care for people living with HIV infection in sub-Saharan Africa: a systematic review.. J Int AIDS Soc.

[11] Mugglin C, Estill J, Wandeler G, Bender N, Egger M, Gsponer T (2012). Loss to programme between HIV diagnosis and initiation of antiretroviral therapy in sub-Saharan Africa: systematic review and meta-analysis.. Trop Med Int Health.

[12] Somi G, Keogh S, Todd J, Kilama B, Wringe A, Van Den Hombergh J (2012). Low mortality risk but high loss to follow-up among patients in the Tanzanian national HIV care and treatment programme.. Trop Med Int Health.

[13] National AIDS Control Programme - The United Republic of Tanzania Ministry of Health and Social Welfare Implementation of HIV/AIDS Care and Treatment Services in Tanzania - Report Number 4. 2016. Available: http://nacp.go.tz/site/download/Report_number_4.pdf 2016. Accessed: 10-Sept-2018.

[14] Fox MP, Rosen S (2015). Retention of adult patients on antiretroviral therapy in low-and middle-income countries: systematic review and meta-analysis 2008–2013.. J Acquir Immune Defic Syndr.

[15] Manyahi J, Jullu BS, Abuya MI, Juma J, Kilama B, Sambu V (2017). Decline in the prevalence HIV among pregnant women attending antenatal clinics in Tanzania, 2001-2011.. Tanzan J Health Res.

[16] Centers for Disease Control and Prevention (CDC) (2013). Differences between HIV-Infected men and women in antiretroviral therapy outcomes-six African countries, 2004-2012.. MMWR Morb Mortal Wkly Rep.

[17] Brinkhof MW, Dabis F, Myer L, Bangsberg DR, Boulle A, Nash D (2008). Early loss of HIV-infected patients on potent antiretroviral therapy programmes in lower-income countries.. Bull World Health Organ.

[18] Moshabela M, Schneider H, Cleary SM, Pronyk PM, Eyles J (2011). Does accessibility to antiretroviral care improve after down-referral of patients from hospitals to health centres in rural South Africa?. Afr J AIDS Res.

[19] Kadobera D, Sartorius B, Masanja H, Mathew A, Waiswa P (2012). The effect of distance to formal health facility on childhood mortality in rural Tanzania, 2005–2007.. Glob Health Action.

[20] Tanser F (2006). Methodology for optimising location of new primary health care facilities in rural communities: a case study in KwaZulu-Natal, South Africa.. J Epidemiol Community Health.

[21] McIntyre D, Thiede M, Birch S (2009). Access as a policy-relevant concept in low-and middle-income countries.. Health Econ Policy Law.

[22] World Health Organization. Scaling up anti-retroviral therapy in resource limited settings. A public health approach. Geneva: World Health Organisation; 2003.

[23] Hallett TB, Eaton JW (2013). A side door into care cascade for HIV-infected patients?. J Acquir Immune Defic Syndr.

[24] Zürcher K, Mooser A, Anderegg N, Tymejczyk O, Couvillon MJ, Nash D (2017). Outcomes of HIV-positive patients lost to follow-up in African treatment programs..

[25] Colasanti J, Kelly J, Pennisi E, Hu YJ, Root C, Hughes D (2016). Continuous retention and viral suppression provide further insights into the HIV care continuum compared to the cross-sectional HIV care cascade.. Clin Infect Dis.

[26] Haber N, Pillay D, Porter K, Bärnighausen T (2016). Constructing the cascade of HIV care: methods for measurement.. Curr Opin HIV AIDS.

