# Supplementary material for: Changes in patterns of retention in HIV care and antiretroviral treatment in Tanzania between 2008 and 2016: an analysis of routinely collected national programme data
Source: J Glob Health. 2019 Mar 30;9(1):010424. doi: 10.7189/jogh.09.010424 (PMC6445500; doi:10.7189/jogh.09.010424)
Supplement: Online Supplementary Document [file jogh-09-010424-s001.pdf]

Mee et al. J Glob Health, 2019;9:010424

Appendix

			**Percentage retained in care at time (95% confidence intervals)**		
**Year of enrolment in care**		**Total Enrolled [1]**	**12 months**	**24 months**	**36 months**
**2008**		67 840	82.8(82.5-83.0)	59.2(58.8-59.5)	46.7(46.3-47.1)
**2009**		78 805	81.5(81.2-81.8)	56.9(56.5-57.2)	44.5(44.2-44.9)
**2010**		78 395	79.8(79.5-80.0)	55.4(55.1-55.8)	44.2(43.9-44.6)
**2011**		69 337	80.2(79.9-80.5)	57.2(56.8-57.5)	45.6(45.2-45.9)
**2012**		70 336	80.6(80.3-80.9)	57.2(56.9-57.6)	45.1(44.8-45.5)
**2013**		87 761	80.5(80.3-80.8)	57.5(57.2-57.8)	46.3(46.0-46.7)
**2014**		102 611	80.4(80.1-80.6)	57.7(57.4-58.0)	-
**2015**		98 120	81.5(81.2-81.7)	-	-
**Overall**		**653 205**	**80.9(80.6-81.1)**	**57.3(56.9-57.6)**	**45.4(45.1-45.8)**
	**Gender**				
**2008**	F	46 333	84.1(83.8-84.4)	60.6(60.1-61.0)	48.1(47.6-48.6)
	M	21 501	79.9(79.3-80.4)	56.1(55.4-56.7)	43.6(43.0-44.3)
**2009**	F	53 918	82.4(82.0-82.7)	57.9(57.5-58.4)	45.7(45.2-46.1)
	M	24 886	79.6(79.1-80.1)	54.6(54.0-55.2)	42.1(41.4-42.7)
**2010**	F	53 611	80.7(80.4-81.1)	56.6(56.2-57.1)	45.4(45.0-45.9)
	M	24 784	77.7(77.2-78.2)	52.8(52.2-53.4)	41.6(40.9-42.2)
**2011**	F	46 830	81.0(80.7-81.4)	58.2(57.8-58.7)	46.5(46.0-46.9)
	M	22 507	78.4(77.8-78.9)	55.0(54.3-55.6)	43.7(43.0-44.3)
**2012**	F	47 619	81.5(81.1-81.8)	58.4(57.9-58.8)	46.0(45.6-46.5)
	M	22 717	78.9(78.4-79.4)	54.9(54.2-55.5)	43.4(42.7-44.0)
**2013**	F	59 869	81.3(81.0-81.6)	58.1(57.7-58.5)	46.6(46.2-47.0)
	M	27 892	78.9(78.5-79.4)	56.2(55.6-56.8)	45.7(45.1-46.2)
**2014**	F	70 983	80.7(80.4-81.0)	57.8(57.4-58.2)	-
	M	31 628	79.6(79.2-80.1)	57.4(56.9-58.0)	-
**2015**	F	66 031	81.8(81.5-82.1)	-	-
	M	32 089	80.9(80.4-81.3)	-	-
**Overall**	**F**	**445 194**	**81.6(81.3-81.9)**	**58.2(57.7-58.6)**	**46.4(45.9-46.8)**
	**M**	**208 004**	**79.3(78.8-79.8)**	**55.4(54.8-56.0)**	**43.4(42.7-44.0)**

Table S1a – Numbers and percentages of each annual enrolment cohort retained at 12, 24 and 36 months for the entire cohort and stratified by sex

[1] Where data was missing for sex and age these individuals are omitted from annual totals

			Percentage retained in care at time (95% confidence intervals)		
**Year of enrolment in care**		**Total Enrolled [1]**	**12 months**	**24 months**	**36 months**
	**Age at enrolment**				
**2008**	15-24	6807	81.2(80.3-82.1)	52.0(50.8-53.2)	38.7(37.5-39.8)
	25-34	25 392	82.8(82.3-83.3)	58.5(57.9-59.1)	46.2(45.6-46.8)
	35-49	28 055	83.2(82.8-83.6)	61.5(61.0-62.1)	49.0(48.4-49.6)
	>49	7586	82.3(81.5-83.2)	58.9(57.8-60.0)	46.9(45.8-48.0)
**2009**	15-24	8517	79.4(78.5-80.3)	49.6(48.6-50.7)	36.2(35.2-37.3)
	25-34	29 497	81.1(80.6-81.5)	55.6(55.0-56.1)	43.1(42.5-43.7)
	35-49	31 954	82.2(81.8-82.7)	59.4(58.9-60.0)	47.4(46.9-48.0)
	>49	8837	82.2(81.3-82.9)	59.2(58.1-60.2)	46.8(45.7-47.8)
**2010**	15-24	8563	77.1(76.2-78.0)	47.7(46.6-48.7)	35.6(34.6-36.6)
	25-34	28 760	79.2(78.7-79.7)	54.2(53.7-54.8)	42.8(42.3-43.4)
	35-49	31 917	80.8(80.4-81.2)	57.9(57.4-58.5)	47.1(46.6-47.7)
	>49	9155	80.5(79.6-81.3)	57.6(56.6-58.6)	46.4(45.4-47.4)
**2011**	15-24	7416	76.8(75.9-77.8)	49.0(47.9-50.1)	35.0(33.9-36.0)
	25-34	24 981	79.9(79.4-80.4)	55.5(54.9-56.1)	43.5(42.9-44.1)
	35-49	28 541	81.4(81.0-81.9)	60.2(59.7-60.8)	49.3(48.7-49.9)
	>49	8399	79.7(78.8-80.6)	58.9(57.9-60.0)	48.5(47.4-49.6)
**2012**	15-24	7518	76.1(75.1-77.1)	46.8(45.7-47.9)	32.5(31.5-33.6)
	25-34	24 985	80.1(79.6-80.6)	55.3(54.6-55.9)	42.2(41.6-42.8)
	35-49	29 089	81.9(81.4-82.3)	60.6(60.0-61.1)	49.7(49.1-50.2)
	>49	8744	81.9(81.1-82.7)	60.7(59.7-61.7)	49.3(48.3-50.4)
**2013**	15-24	10 145	76.4(75.5-77.2)	46.2(45.3-47.2)	32.5(31.6-33.4)
	25-34	30 657	79.5(79.0-79.9)	54.5(53.9-55.1)	42.5(42.0-43.1)
	35-49	35 456	82.2(81.8-82.6)	62.0(61.5-62.5)	51.9(51.4-52.4)
	>49	11 503	81.8(81.1-82.5)	61.5(60.6-62.3)	51.5(50.6-52.4)
**2014**	15-24	13 841	75.5(74.8-76.2)	45.6(44.7-46.4)	-
	25-34	35 817	79.2(78.7-79.6)	55.1(54.6-55.6)	-
	35-49	39 669	82.4(82.1-82.8)	62.4(61.9-62.9)	-
	>49	13 284	82.6(82.0-83.2)	63.2(62.4-64.0)	-
**2015**	15-24	12 789	76.5(75.8-77.2)	-	-
	25-34	32 705	80.4(79.9-80.8)	-	-
	35-49	38 934	83.4(83.0-83.8)	-	-
	>49	13 692	83.3(82.7-83.9)	-	-
**Overall**	**15-24**	**75 596**	**77.1(76.3-77.9)**	**47.8(46.7-48.8)**	**34.9(33.9-36.0)**
	**25-34**	**232 794**	**80.2(79.7-80.7)**	**55.5(54.9-56.0)**	**43.3(42.8-43.9)**
	**35-49**	**263 615**	**82.2(81.8-82.6)**	**60.6(60.1-61.2)**	**49.1(48.6-49.7)**
	**>49**	**81 200**	**81.9(81.2-82.6)**	**60.3(59.3-61.2)**	**48.4(47.4-49.4)**

Table S1b - Numbers and percentages of each annual enrolment cohort retained at 12, 24 and 36 months for the entire stratified by age

[1] Where data was missing for sex and age these individuals are omitted from annual totals

Year of ART initiation	Gender	Total initiated on treatment [1]	Percentage retained on treatment at time (95% confidence intervals) 12 months	24 months	36 months
**2008**		34 584	84.6(84.3-85.0)	65.5(65.1-66.0)	53.5(53.0-54.0)
**2009**		47 869	84.4(84.1-84.8)	64.3(63.9-64.8)	52.3(51.8-52.7)
**2010**		52 041	83.0(82.7-83.3)	63.0(62.6-63.4)	52.3(51.8-52.7)
**2011**		51 780	84.4(84.1-84.8)	65.5(65.1-65.9)	54.4(54.0-54.9)
**2012**		66 432	85.1(84.9-85.4)	66.0(65.7-66.4)	54.4(54.0-54.7)
**2013**		83 723	84.4(84.2-84.7)	64.5(64.1-64.8)	53.6(53.2-53.9)
**2014**		107 030	82.7(82.5-83.0)	61.7(61.4-62.0)	-
**2015**		100 616	83.6(83.4-83.9)	-	-
**Overall**		**544 075**	**83.9(83.6-84.2)**	**64.0(63.7-64.4)**	**53.5(53.0-53.9)**
	**Gender**				
**2008**	F	22 510	85.8(85.4-86.2)	67.2(66.7-67.8)	55.2(54.6-55.8)
	M	12 071	82.3(81.7-83.0)	62.2(61.4-63.0)	50.2(49.3-51.0)
**2009**	F	31 271	85.4(85.0-85.8)	65.6(65.1-66.1)	53.9(53.3-54.4)
	M	16 597	82.6(82.0-83.1)	61.9(61.1-62.6)	49.1(48.4-49.9)
**2010**	F	34 334	84.3(83.9-84.6)	64.7(64.2-65.2)	54.2(53.6-54.7)
	M	17 706	80.6(80.0-81.1)	59.5(58.7-60.2)	48.5(47.8-49.3)
**2011**	F	34 272	85.5(85.1-85.9)	67.3(66.8-67.8)	56.2(55.7-56.8)
	M	17 508	82.3(81.7-82.9)	61.9(61.2-62.7)	50.8(50.1-51.6)
**2012**	F	44 647	86.2(85.8-86.5)	67.7(67.3-68.2)	56.0(55.5-56.4)
	M	21 785	83.0(82.4-83.5)	62.5(61.8-63.1)	51.0(50.3-51.7)
**2013**	F	57 384	85.4(85.1-85.7)	65.5(65.1-65.9)	54.3(53.9-54.7)
	M	26 339	82.4(81.9-82.8)	62.3(61.7-62.8)	51.9(51.2-52.5)
**2014**	F	77 489	82.8(82.5-83.0)	61.4(61.1-61.8)	-
	M	29 541	82.7(82.2-83.1)	62.5(61.9-63.0)	-
**2015**	F	69 259	83.7(83.5-84.0)	-	-
	M	31 357	83.3(82.9-83.8)	-	-
**Overall**	**F**	**371 166**	**84.6(84.2-84.9)**	**65.0(64.6-65.5)**	**54.9(54.4-55.4)**
	**M**	**172 904**	**82.5(82.0-83.0)**	**61.9(61.2-62.5)**	**50.4(49.7-51.2)**

Table S2a – Numbers and percentages of each annual ART initiation cohort retained at 12, 24 and 36 months after ART initiation for the entire cohort and stratified by sex

[1] Where data was missing for sex and age these individuals are omitted from annual totals

			Percentage retained on treatment at time (95% confidence intervals)		
**Year of ART initiation**		**Total initiated on treatment [1]**	**12 months**	**24 months**	**36 months**
	**Age at enrolment**				
**2008**	15-24	2252	82.9(81.4-84.2)	58.6(56.8-60.4)	44.9(43.0-46.7)
	25-34	12 073	84.3(83.7-84.9)	64.7(64.0-65.5)	52.3(51.5-53.1)
	35-49	15 833	85.3(84.8-85.8)	67.4(66.8-68.1)	55.7(55.0-56.4)
	>49	4426	84.1(83.1-85.1)	64.3(63.0-65.5)	53.2(51.9-54.5)
**2009**	15-24	3351	81.7(80.4-83.0)	57.8(56.1-59.5)	43.9(42.3-45.6)
	25-34	16 613	83.8(83.2-84.3)	62.7(62.0-63.5)	50.8(50.1-51.6)
	35-49	21 696	85.2(84.7-85.6)	66.1(65.5-66.7)	54.2(53.5-54.8)
	>49	6209	85.0(84.1-85.9)	65.7(64.6-66.9)	53.7(52.4-54.9)
**2010**	15-24	3683	79.9(78.5-81.2)	55.6(53.9-57.2)	44.0(42.3-45.6)
	25-34	17 550	82.2(81.6-82.7)	61.2(60.5-62.0)	50.1(49.3-50.8)
	35-49	23 672	83.9(83.5-84.4)	64.8(64.2-65.4)	54.6(53.9-55.2)
	>49	7136	83.6(82.7-84.4)	64.8(63.7-65.9)	54.0(52.9-55.2)
**2011**	15-24	3594	80.2(78.8-81.5)	56.7(55.1-58.4)	42.2(40.5-43.9)
	25-34	17 354	84.5(83.9-85.0)	64.1(63.4-64.8)	52.4(51.6-53.1)
	35-49	23 533	85.1(84.7-85.6)	67.6(67.0-68.2)	57.2(56.5-57.8)
	>49	7299	84.2(83.3-85.0)	66.3(65.2-67.3)	56.2(55.1-57.4)
**2012**	15-24	4852	80.0(78.8-81.1)	54.8(53.4-56.2)	40.4(39.0-41.8)
	25-34	22 385	84.5(84.0-85.0)	64.2(63.6-64.8)	51.5(50.8-52.1)
	35-49	29 943	86.2(85.8-86.6)	68.5(68.0-69.0)	57.9(57.3-58.5)
	>49	9252	85.9(85.2-86.6)	68.2(67.2-69.1)	56.8(55.8-57.8)
**2013**	15-24	7664	79.0(78.1-79.9)	52.4(51.3-53.5)	38.6(37.5-39.7)
	25-34	28 350	83.7(83.3-84.1)	61.8(61.2-62.4)	49.9(49.3-50.5)
	35-49	35 925	85.9(85.6-86.3)	68.2(67.7-68.7)	58.3(57.8-58.8)
	>49	11 784	85.0(84.3-85.6)	67.2(66.3-68.0)	57.3(56.4-58.2)
**2014**	15-24	14 250	76.6(75.9-77.3)	48.0(47.2-48.9)	-
	25-34	38 627	81.4(81.0-81.8)	58.5(58.0-59.0)	-
	35-49	40 954	85.1(84.8-85.5)	67.2(66.7-67.6)	-
	>49	13 199	85.8(85.1-86.3)	68.5(67.7-69.3)	-
**2015**	15-24	12 115	77.5(76.8-78.3)	-	-
	25-34	33 231	82.3(81.8-82.7)	-	-
	35-49	40 893	85.8(85.5-86.2)	-	-
	>49	14 377	85.6(85.0-86.2)	-	-
**Overall**	**15-24**	**51 761**	**78.6(77.6-79.5)**	**52.6(51.4-53.9)**	**41.5(40.0-43.0)**
	**25-34**	**186 183**	**83.1(82.6-83.5)**	**61.8(61.2-62.5)**	**51.0(50.3-51.7)**
	**35-49**	**23 2449**	**85.4(85.0-85.8)**	**67.2(66.7-67.8)**	**56.6(56.0-57.2)**
	**>49**	**73 682**	**85.1(84.3-85.8)**	**66.9(65.9-67.8)**	**55.6(54.6-56.7)**

Table S2b - Numbers and percentages of each annual ART initiation cohort retained at 12, 24 and 36 months after ART initiation stratified by age

[1] Where data was missing for sex and age these individuals are omitted from annual totals

		Rate ratio [1]	95% CI
**Year that clinic first submitted data to the CTC3 database**	**2005**	1	-
	**2006**	0.97	0.96 - 0.99
	**2007**	0.95	0.93 - 0.96
	**2008**	0.96	0.95 - 0.97
	**2009**	0.88	0.87 - 0.90
	**2010**	0.99	0.97 - 1.01
	**2011**	0.96	0.94 - 0.98
	**2012**	0.99	0.97 - 1.01
	**2013**	0.92	0.90 - 0.94
	**2014**	0.95	0.92 - 0.98
	**2015**	0.79	0.72 - 0.88

Table S3 – Bivariate Cox regression analysis of the effect of the year that data was first submitted by a clinic to the CTC3 database on the rate of loss to follow-up from care.

[1] The rate ratio compares the rates loss to follow-up after enrolment in care between the baseline category and each subsequent stratum.
